# Direct profiling of the phospholipid composition of adult *Caenorhabditis elegans* using whole-body imaging mass spectrometry

**DOI:** 10.1007/s00216-015-8932-7

**Published:** 2015-08-27

**Authors:** Saira Hameed, Koji Ikegami, Eiji Sugiyama, Shoko Matsushita, Yoshishige Kimura, Takahiro Hayasaka, Yuki Sugiura, Noritaka Masaki, Michihiko Waki, Isao Ohta, Md Amir Hossen, Mitsutoshi Setou

**Affiliations:** Department of Cell Biology and Anatomy, Hamamatsu University School of Medicine, 1-20-1 Handayama, Higashi-ku, Hamamatsu, Shizuoka 431-3192 Japan; Research Equipment Center, Hamamatsu University School of Medicine, 1-20-1 Handayama, Higashi-ku, Hamamatsu, Shizuoka 431-3192 Japan; Faculty of Health Sciences, Health Innovation and Technology Center, Hokkaido University, Kita 12 Nishi 5, Kita-ku, Sapporo, Hokkaido 060-0812 Japan; Department of Biochemistry, School of Medicine, Keio University, Shinjuku-ku, Tokyo, 160-8582 Japan

**Keywords:** *Caenorhabditis elegans*, Cuticle, Exoskeleton, Freeze-cracking, Matrix-assisted laser desorption/ionization-imaging mass spectrometry, Phosphatidylcholine, Phosphatidylinositol

## Abstract

A protocol for the direct analysis of the phospholipid composition in the whole body of adult soil nematode, *Caenorhabditis elegans* (*C. elegans*), was developed, which combined freeze-cracking of the exoskeletal cuticle and matrix-assisted laser desorption/ionization-imaging mass spectrometry (MALDI-IMS). Biomolecules in the *m*/*z* range from 700 to 900 were more effectively detected in the freeze-cracked than from simple frozen adult nematode bodies. Different distribution of biomolecules was observed in a nematode body when the matrix was applied with a sublimation deposition method. The whole-body IMS technique was applied on genetically deficient mutant *C. elegans* to combine whole-body lipidomics and genetics, by comparing the fatty acid compositions, especially of the phosphatidylcholine (PC) species, between the wild-type and *fat-1* mutants, which lack the gene encoding an *n-3* fatty acid desaturase. A significant reduction of PC(20:5/20:5) and PC(20:4/20:5) and a marked increase of PC(20:4/20:4), PC(20:3/20:4), and PC(20:3/20:3) were detected in the *fat-1* mutants in positive ion mode. In addition, phospholipid compositions other than PCs were analyzed in negative ion mode. A loss of a possible phosphatidylinositol (PI) with 18:0/20:5 and a compensative accumulation of putative PI(18:0/20:4) were detected in the *fat-1* mutants. In conclusion, the whole-body MALDI-IMS technique is useful for the profiling of multiple biomolecules in *C. elegans* in both intra- and inter-individual levels.

**Electronic supplementary material:**

The online version of this article (doi:10.1007/s00216-015-8932-7) contains supplementary material, which is available to authorized users.

## Introduction

Recent advances in mass spectrometry have enabled the direct analyses of biomolecules in tissue samples without any target-specific labeling [1, 2]. Matrix-assisted laser desorption/ionization-imaging mass spectrometry (MALDI-IMS) can be used for the analyses of the spatial distribution of various biomolecules, which range from small metabolites to lipids, peptides, and intact proteins, in tissue sections [3–6]. The state of the art MALDI-IMS technique has been used for the investigation of molecular distributions in mammalian tissues [7], including samples of diseased human tissues [8–10]. It has also been used for label-free non-targeted analyses of biomolecules in various species [11], such as microbes [12], plants [13], parasites [14], arthropods, including crustaceans such as the giant tiger prawn (*Penaeus monodon*) [15], and insects such as the fruit fly (*Drosophila melanogaster*) [16, 17].

The soil nematode, *Caenorhabditis elegans* (*C. elegans*), is a common model organism and is extensively used in life science research [18]. *C. elegans* has a wide range of advantages for experimental research [19]. The short life span of minimally 3 days and the capability of being frozen enable a number of iteration of experimental tests in a short time [18]. These allow quick forward genetics, combined with the ease of genetic manipulations [20]. The body structure with multiple organs composed of the fixed number of cells (~1000 somatic cells) [18], with well-characterized cell fate is highly powerful to developmental biology. Given these advantages in multiple directions, *C. elegans* has a big potential to provide a powerful platform for “Integrating-Omics,” in which genetics, transcriptomics, proteomics, lipidomics, and metabolomics are combined to find new insights [21], opening a new era of life sciences. *C. elegans* also begins to be used in applied sciences, such as drug discovery or screening [22].

Some studies have investigated the metabolomic profiling of genetically deficient mutant nematodes [23–28]. *C. elegans* has a balloon-like body with high osmotic pressure that is enclosed by an exoskeleton consisting of a tough impermeable cuticle [29]. This has hindered the direct detection and analysis of the biomolecules contained inside. Thus, the components of nematode bodies have been extracted in most metabolomics analyses. The very thick and rigid exoskeleton was thought to inhibit a direct “whole-body” IMS, as the cuticle layer of plants should be bypassed using vibratome sectioning before the IMS analysis of molecules beneath their cuticles [30]. Moreover, the cryosectioning of exoskeletal organisms requires special handling, which is time-consuming and requires well-designed equipment [16]. Thus, the development of a facile sample preparation for the analysis of the biomolecules using the whole-body IMS of *C. elegans* has been desired.

Our aim was to establish a facile protocol for the whole-body MALDI-IMS of adult *C. elegans*. To accomplish this, we combined a freeze-cracking technique with MALDI-IMS and were able to successfully analyze nematodes to visualize biomolecules in an individual nematode level. We further combined the whole-body MALDI-IMS to genetics through the comparison of the wild-type and *fat-1* mutants and succeeded in detecting significant differences in the fatty acid compositions of the phosphatidylcholine (PC) and phosphatidylinositol (PI) species between the two genetically different nematode lines.

## Materials and methods

### Chemicals

Methanol (MeOH), ethanol (EtOH), chloroform (CHCl_3_), ultrapure water, and potassium acetate (CH_3_COOK) were purchased from Wako Pure Chemical Industries (Osaka, Japan). Calibration standard peptides (human bradykinin and angiotensin II) were purchased from Bruker Daltonics (Billerica, MA, USA). 2,5-Dihydroxybenzoic acid (DHB) was purchased from Bruker Daltonics (Billerica, MA, USA) or Sigma-Aldrich (St. Louis, MO, USA). 9-Aminoacridine hemihydrate (9-AA) was purchased from Acros Organics (NJ, USA).

### Nematodes

*C. elegans* strains were grown at 20 °C under standard conditions [18] on nematode growth medium (NGM) agar plates (0.3 % NaCl, 0.25 % Bacto Peptone, 1.5 % agar, 0.0005 % cholesterol, 1 mM CaCl_2_, 1 mM MgSO_4_, 25 mM potassium phosphate buffer [pH 6.0]), which were seeded with the OP50 *Escherichia coli* strain as a food source. The wild-type strain (Bristol N2) and *fat-1* mutant (BX24: *fat-1(wa9) IV*) of *C. elegans* were obtained from the Caenorhabditis Genetics Center (Minneapolis, MN, USA). A synchronous culture of *C. elegans* was obtained by bleaching the nematodes 3 days before observation. Only adult nematodes were used for the MALDI-IMS and liquid chromatography-electrospray ionization-tandem mass spectrometry (LC-ESI-MS/MS) analyses.

### Sample preparation for MALDI-IMS

**Fig. 1:**
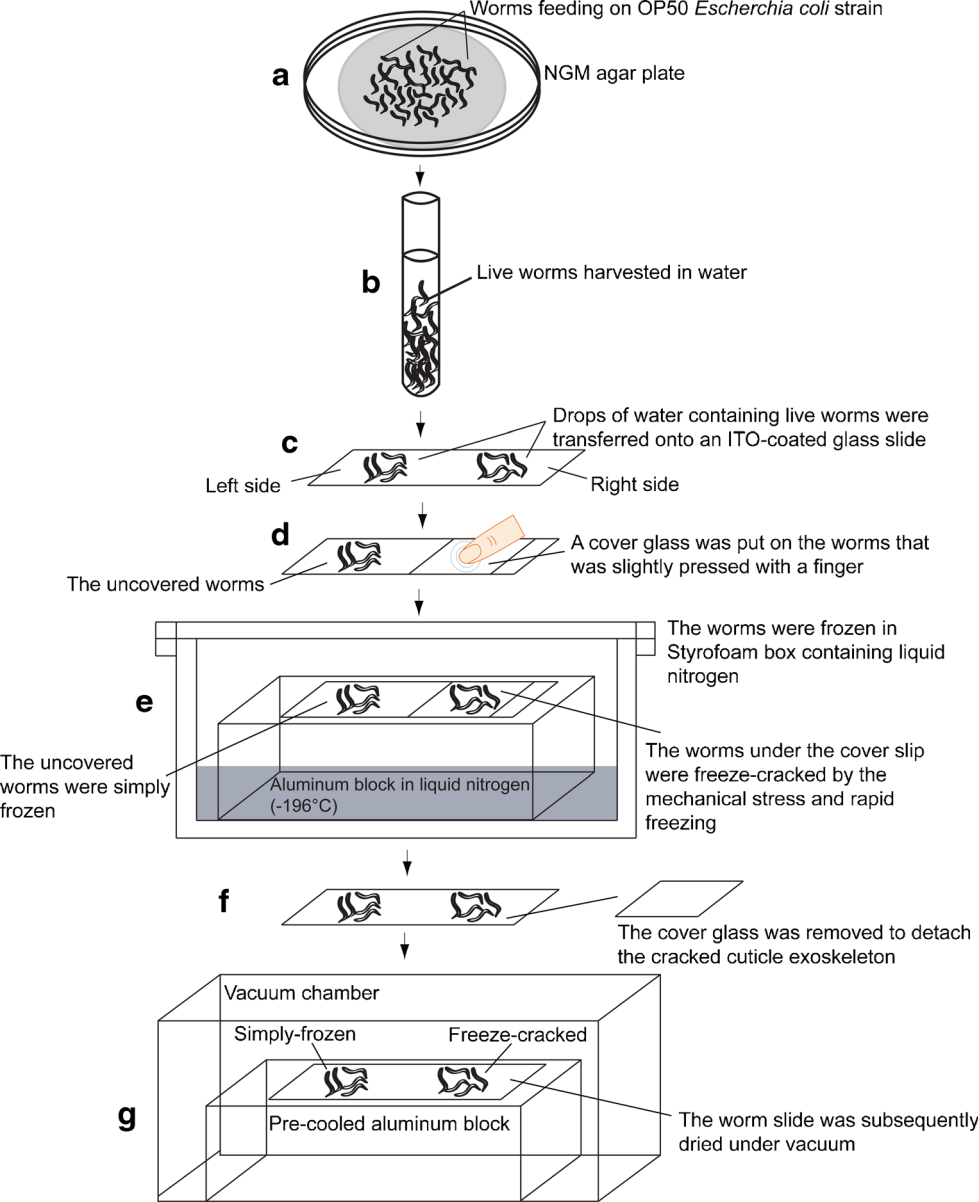
Workflow for whole-body MALDI-IMS analyses of adult *C. elegans*. **a**, **b** Harvesting live nematodes from a nematode growth medium (NGM) agar plate into a glass tube by suspension in water. **c** Seeding the nematodes onto an ITO-coated glass slide. **d** Applying a cover glass on a nematode subset and pressing them for freeze-cracking (on the right side of the glass slide). The other subset of live nematodes was left untreated. **e** Rapid freezing of the nematodes on a liquid nitrogen-cooled aluminum block. Through this process, the nematodes that were covered by the coverslip were freeze-cracked, whereas the others were simply frozen. **f** Removing the coverslip to detach the cuticle exoskeleton from the nematodes. **g** Vacuum drying of the nematodes on a pre-cooled aluminum block

The nematodes that were grown on the surface of the NGM agar plates were harvested by thoroughly washing with water (Fig. 1a), and the live nematodes were transferred into a glass tube using a Pasteur pipette (Fig. 1b). Subsequently, water droplets containing live nematodes were transferred onto indium-tin-oxide (ITO)-coated glass slides (Bruker Daltonics) (Fig. 1c). The freeze-cracking method involved covering the nematode containing water droplets with a cover glass that was lightly pressed with a finger to immobilize the nematodes and then to make direct contact with their surfaces (Fig. 1d). The sample slide was then rapidly frozen on an aluminum block at liquid nitrogen temperature (−196 °C) (Fig. 1e). The aluminum block allowed for a faster heat transfer, which generated a temperature gradient across *C. elegans* because of their cylindrical morphology. The cover glass was quickly detached to remove the cracked cuticle exoskeletons (Fig. 1f). The sample slide was then dried under vacuum for ~1 h (Fig. 1g).

To compare the freeze-cracked and simple frozen nematodes, water droplets containing live nematodes were transferred to the left and right sides of an ITO-coated glass slide. The left side was used for the simple frozen nematodes, whereas the right was used for the freeze-cracked nematodes (Fig. 1d). Simple frozen nematodes were not subjected to any cracking treatment but were frozen and dried simultaneously with cracked ones (Fig. 1e–g).

### Scanning electron microscopy

Scanning electron microscopy (SEM) was performed as previously reported [31, 32]. Simple frozen and freeze-cracked nematode specimens were coated with osmium tetroxide (OsO_4_) using a plasma multi coater model PMC-5000 (Meiwa, Japan). The SEM observation of nematodes was performed using a Hitachi S-4800 field emission scanning electron microscope at an acceleration voltage of 1.0 kV and an emission current of 10 μA. The vacuum level in the observation chamber was 10^−5^–10^−7^ Pa, and the working distance was 8.0 mm.

### Matrix application

DHB was chosen to detect PC in positive ion mode, since it is most commonly used for MALDI-MS and MALDI-IMS with high vacuum chamber [33–35]. 9-AA was used to detect PI in negative ion mode, since it is most common for lipid analyses in negative ion mode of MALDI-MS and MALDI-IMS [36, 37]. To extract and co-crystallize the analytes, the nematode samples were spray-coated with DHB solution (50 mg/mL in 70 % MeOH containing 20 mM CH_3_COOK) for analyses in positive ion mode, or 9-AA solution (10 mg/mL in 70 % EtOH) for analyses in negative ion mode on an ITO-coated glass slide using a 0.2-mm caliber nozzle airbrush (Procon Boy FWA Platinum; Mr. Hobby, Tokyo, Japan). Approximately 2 mL of DHB or 3 mL of 9-AA solutions were sprayed over 15 to 20 min. The airbrush was moved right to left, top to bottom, and vice versa, for around 1200 times, while maintaining a distance of 10 cm between the nozzle and nematode tissue surfaces. After the matrix application, the slide was incubated in desiccator for around 5 min. The humidity of room was maintained under 25 % at 23 °C. The glass slide was observed by a microscope to confirm whether the matrix layer uniformly covered the nematode sample surfaces.

The nematode samples were also subjected to matrix application by sublimation deposition method, with 600 mg of DHB sublimated at 170 °C for the deposition thickness to reach 1.5 μm, using iMLayer (Shimadzu, Japan).

### MALDI-IMS

MALDI-IMS was performed using an ultraflex II TOF/TOF instrument (Bruker Daltonics), equipped with a Smartbeam-II Nd:YAG 355 nm laser, with 25 μm in raster scan pitch. The laser frequency was 200 Hz, and the data were acquired using an ion source voltage of 25 kV and a reflector voltage of 26.30 kV in the positive ion reflectron mode. Calibration of the MS was performed using DHB ([M + H]^+^, *m*/*z* 155.03), human bradykinin fragment 1–7 ([M + H]^+^, *m*/*z* 757.40), and human angiotensin II ([M + H]^+^, *m*/*z* 1046.54). In negative ion mode, the laser frequency was 100 Hz, and the data were acquired using an ion source voltage of 20.11 kV and a reflector voltage of 21.07 kV. The data acquisition areas, over which the spectra were measured, were set by tracing the outline of the well-cracked nematodes. These were characterized by the observation of the nematode bodies and the region immediately outside them. The mass spectra were acquired by averaging the signals from 500 laser pulses per sample measurement point, and the mass measurement range was set to *m*/*z* 700–1000. The nematode specimens were automatically raster scanned using flexControl (ver. 3 or 3.4) and flexImaging (ver. 2.1 or 4.0) software (Bruker Daltonics). The acquired raw mass spectra were normalized to the total ion current (TIC). The images of the detected molecular ions were constructed using the flexImaging (2.1 and 4.0) software. Six simple frozen and six freeze-cracked nematodes were analyzed to evaluate the effectiveness of the exoskeleton removal. Four nematodes were analyzed to examine whether biomolecules were retained in nematode bodies. Six wild-type and *fat-1* mutant nematodes were analyzed in positive ion mode. Eight wild-type and *fat-1* mutant nematodes were analyzed in negative ion mode. The data were presented as plots for each nematode and the mean of the signal intensities acquired from the multiple samples.

### Liquid chromatography-electrospray ionization-tandem mass spectrometry (LC-ESI-MS/MS)

Phospholipids of the harvested wild-type and *fat-1* mutants were extracted with Folch method [38], and they were analyzed using LC-ESI-MS/MS with a 4000Q-TRAP triple quadrupole linear ion trap mass spectrometer (AB SCIEX, Framingham, MA, USA) equipped with an ACQUITY ultra-performance liquid chromatography system (Waters, Milford, MA, USA). An ACQUITY UPLC BEH C18 column (2.1 × 50 mm, i.d., 1.7 mm particles; Waters) was connected to a guard column (2.1 × 5 mm; Waters), and the temperature of column oven was maintained at 40 °C [9]. The mobile phase consisted of a gradient of two solvent mixtures. Solvent A was composed of acetonitrile, MeOH, and water (19:19:2 *v*/*v*/*v*), containing formic acid (0.1 vol.%) and ammonia (0.028 vol.%). Solvent B was composed of isopropanol containing formic acid (0.1 vol.%) and ammonia (0.028 vol.%). A gradient elution using solvents A and B was performed at a flow rate of 0.40 mL/min. To profile the molecular species of the specific phospholipid classes, a precursor ion scanning for the polar head groups of the PCs and sphingomyelins (SMs) (*m*/*z* = 184) was performed using the positive ion detection mode of the 4000Q-TRAP instrument. Fragment ions were generated through collision-induced-dissociation (CID) [39]. The optimal collision energy was determined by the preliminarily analysis of PC(16:0/18:1), which was used as a standard lipid. The PC molecular species detected were assigned using their *m*/*z* values and the relative retention times of PC molecular species that were previously described [9, 34, 39], or by referring to the online database, “The Human Metabolome Database (HMDB)” (http://www.hmdb.ca/spectra/ms/search), and the total fatty acid composition of *C. elegans* [23–25] (Table 1).

**Table 1:** Molecular weights of the phosphatidylcholine species in *C. elegans*

PC species	MW	H^+^ Adduct	K^+^ Adduct	ID
PC(20:5/20:5)	825.5	826.5	864.5	HMDB08511
PC(20:4/20:5)	827.5	828.5	866.5	HMDB08478
PC(20:4/20:4)	829.5	830.5	868.5	HMDB08476
PC(20:3/20:4)	831.5	832.5	870.5	HMDB08379
PC(20:3/20:3)	833.5	834.5	872.5	HMDB08377

http://www.hmdb.ca/spectra/ms/search

Further analyses of fatty acid compositions in the PC species of interest were performed by a hybrid quadrupole-Orbitrap mass spectrometer (Q Exactive; Thermo Scientific, Waltham, MA, USA), with the mass resolution of 5 ppm. Separation of the molecular species was carried out using Agilent1100 series HPLC System (Agilent Technologies, Germany) equipped with Acclaim™ 120 C18 column (2.1 × 150 mm, i.d., 3 μm particles; Thermo Scientific). The temperature of the column oven was maintained at 50 °C. The injection volume was 5.0 μL. The temperature of the sample tray was kept at 10 °C. Solvent A was composed of water, acetonitrile, MeOH (2:1:1, *v*/*v*/*v*), containing ammonium formate (5 mM) and formic acid (0.1 vol.%). Solvent B was composed of acetonitrile, isopropanol (1:9, *v*/*v*), containing ammonium formate (5 mM) and formic acid (0.1 vol.%). A gradient elution using solvents A and B was performed at a flow rate of 0.30 mL/min for 50 min from the initial composition (A/B: 80/20, *v*/*v*) to the final composition (A/B: 0/100 vol.%) with a linear gradient. MS and MS/MS analyses were performed in both positive and negative ion modes. MS spectra were acquired in the range of *m*/*z* 700–900. MS/MS spectra near the peak top of interested peaks were acquired with a targeted MS/MS method. The target mass-resolving power at *m*/*z* 200 was set to 70,000 for both MS and MS/MS analyses. The isolation window for MS/MS was set to 0.4 *m*/*z*. The temperature of ion source heater was set to 350 °C, and the capillary temperature was at 250 °C. The ion spray voltage was set to 3.5 kV for both ion modes. Maximum injection time was set to 100 ms for both MS and MS/MS analyses. The automatic gain control target was set to 1 × 10^7^ for MS and 2 × 10^5^ for MS/MS. The normalized collision energy for MS/MS was set to 30 %. Extracted ion chromatograms (EICs) were generated within a theoretical value of ±5 ppm for PC species of interest.

## Results

### Relative effectiveness of the freeze-cracking method in the direct detection of multiple biomolecules

**Fig. 2:**
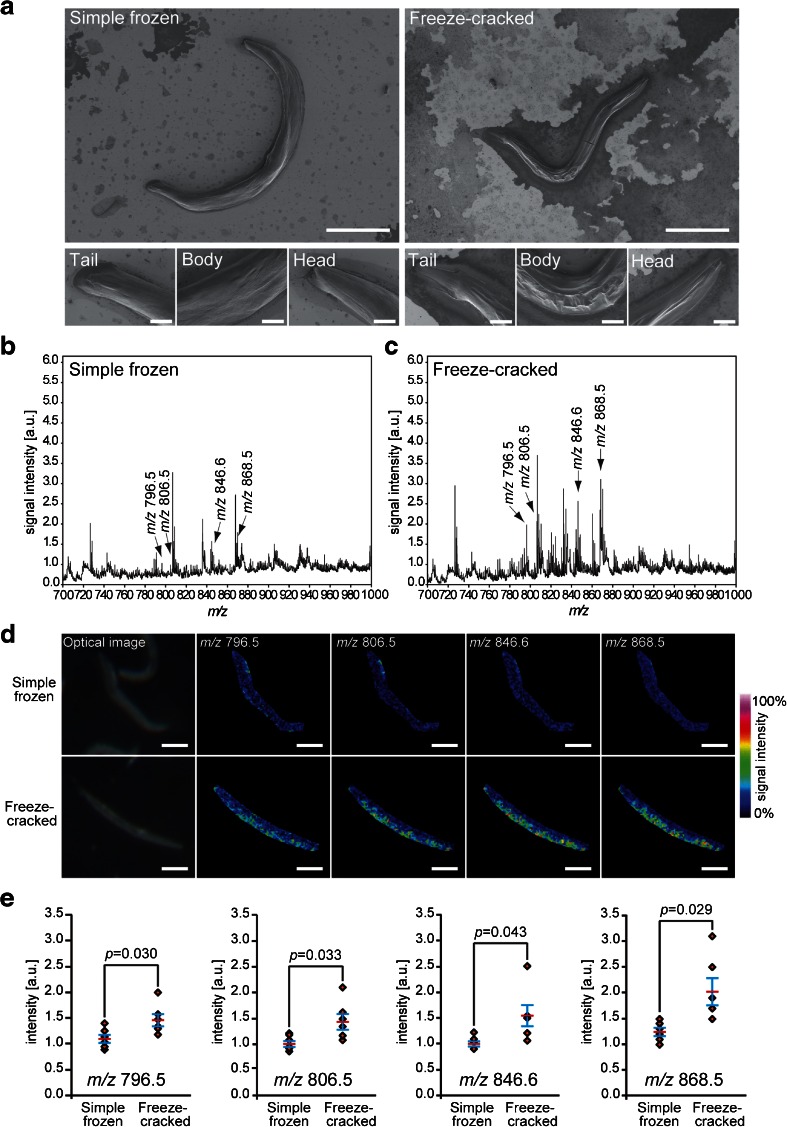
Freeze-cracked nematode bodies yield stronger signals. **a** Scanning electron microscopy images of simple frozen and freeze-cracked *C. elegans*. Scale bars: 200 μm in low magnification (*top*), 25 μm in high magnification (*bottom*). **b**, **c** Averaged mass spectra ranging from *m*/*z* 700 to 1000 that were detected in the whole body of **b** simple frozen and **c** freeze-cracked *C. elegans.* Four of the major mass peaks at *m*/*z* 796.5, 806.5, 846.6, and 868.5 were selected to depict the thermal color scale images. **d** Optical images and thermal color scale images of the four selected molecules on the nematode bodies. Scale bar: 200 μm. Color scale: *deep blue*, faint signal; *red-purple*, maximum signal. **e** Quantitative signal intensities on nematode bodies. The data are shown as plots for each nematode (*diamonds*; *n* = 6) and mean (*red line*) ± SD (*blue lines*). The *p* values were calculated using a *t* test

To evaluate the effectiveness of the exoskeleton removal, we performed the comparative analyses of the two procedures, simple freezing and freeze-cracking, in parallel on a glass slide (Fig. 1). To effectively remove the exoskeletal cuticle of *C. elegans* and expose the internal structures of the nematodes, we placed a cover slip on the nematodes and pressed it with a finger before chilling them using a liquid nitrogen-cooled aluminum block (Fig. 1d). We evaluated the effectiveness of this procedure by comparing it with a simple freezing method. We observed the condition of the samples using an SEM. The surface of the freeze-cracked nematode bodies looked highly scabrous with multiple wrinkles (Fig. 2a; right), whereas the simple frozen nematodes had a highly smooth surface, which appeared to retain an intact exoskeletal cuticle (Fig. 2a; left). The microscopy showed that freeze-cracking drastically changed the surface condition of the sample specimens.

We then analyzed the freeze-cracked nematodes using MALDI-IMS and compared the signal intensity of the biomolecules with those from the simple frozen nematodes (Fig. 2b, c). The averaged mass spectra obtained from the freeze-cracked nematodes had increased signal intensity throughout the *m*/*z* range 700–1000 when compared to the simple frozen nematodes (Fig. 2b vs. Fig. 2c). In particular, the molecular ions that were observed between *m*/*z* 700 and 900 were detected with much higher signal intensities from the freeze-cracked nematodes than those from the simple frozen nematodes. We selected four molecules with *m*/*z* 796.5, 806.5, 846.6, and 868.5, respectively (arrows in Fig. 2b, c), to visually compare their signal intensities from the nematode bodies. The signal intensities of these selected molecules increased by >50 % of the maximum signal level (yellow-to-red colors) in some regions of freeze-cracked nematodes, whereas those in the simple frozen nematodes appeared near the noise level (blue-to-cyan colors) (Fig. 2d). We performed further semi-quantitative analyses of the signal intensities of the four molecules. In all four molecules, the signal intensities detected from the freeze-cracked nematodes were significantly higher than those from the simple frozen nematodes (*n* = 6) (Fig. 2e). These results demonstrated that the freeze-cracking method enabled the highly effective direct detection of multiple internal biomolecules, which provided a facile whole-body MALDI-IMS.

### Molecular distribution analyses in nematode bodies by combining the freeze-cracking method with the matrix sublimation

**Fig. 3:**
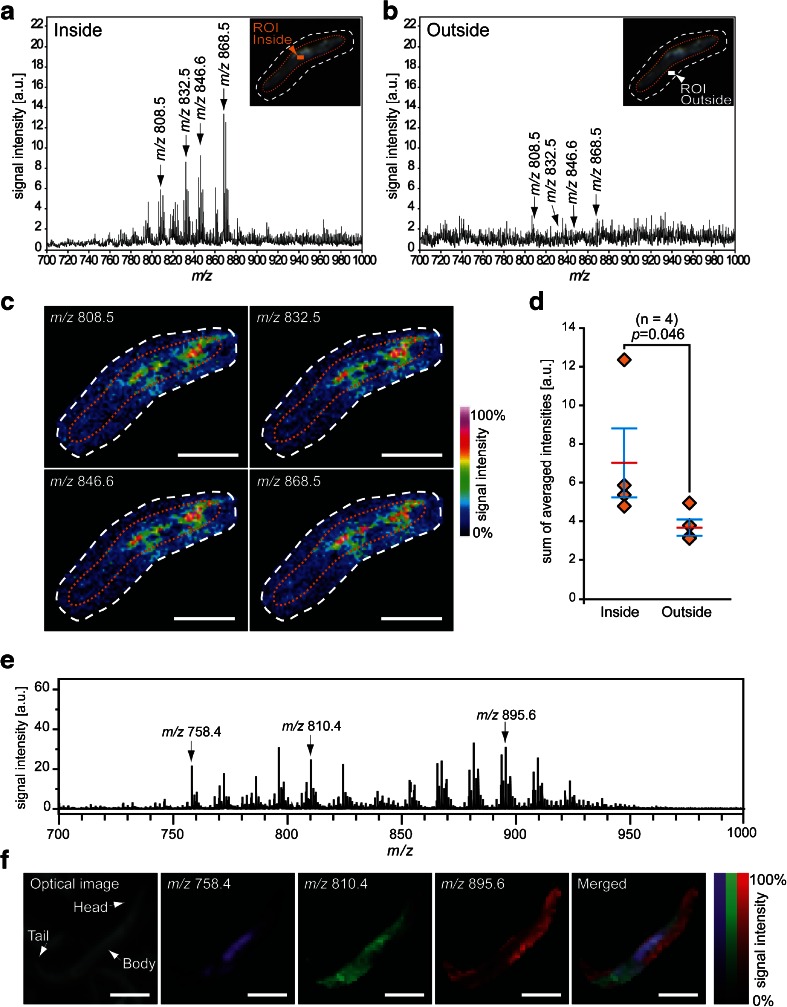
Freeze-cracking method allows analyses of molecular distribution in the nematode body. **a**, **b** Averaged mass spectra detected from the regions of interest **a** inside and **b** outside of the nematode body. Four of the major mass peaks at *m*/*z* 808.5, *m*/*z* 832.5, *m*/*z* 846.6, and *m*/*z* 868.5 were selected to depict thermal color scale images. *Insets* show the outline of nematode body (*orange dotted line*) and scanned area in IMS (*white dotted line*). **c** Thermal color scale images of the selected four molecules. Scale bar: 250 μm. Color scale: *deep blue*, faint signal; *red-purple*, maximal signal. **d** Quantitative signal intensities in the regions of interest. The data are shown as plots for each nematode (*diamonds*; *n* = 4) and mean (*red line*) ± SD (*blue lines*). The *p* values were calculated using a paired *t* test. **e** An averaged mass spectrum detected from freeze-cracked nematode body with DHB matrix applied by sublimation. Three of major mass peaks at *m*/*z* 758.4, 810.4, and 895.6 were selected to depict pseudo color scale images. **f** Pseudo color scale images of the selected three molecules. Scale bar: 250 μm

The nematodes were pressed with a finger during the freeze-cracking procedure (Fig. 1d). This has a potential risk in that the nematode bodies could be punctured during this process, which could result in the delocalization or drift of intra-body ingredients from the nematode body to the surrounding area. Thus, we evaluated whether the biomolecules were retained in the nematode bodies. For this evaluation, we expanded the imaging measurement areas to include the glass surface adjacent to the nematode bodies (Fig. 3a, b; insets) and compared the mass spectra from regions of interest (ROIs) inside and outside of the nematode bodies (Fig. 3a, b; insets). The averaged mass spectrum acquired from the ROI inside of the nematode body (Fig. 3a) has several mass peaks with *m*/*z* values ranging from 750 to 900, whereas that taken from the ROI outside of the nematode body had few significant mass peaks (Fig. 3b).

We selected four of major ion peaks, *m*/*z* values of 808.5, 832.5, 846.6, and 868.5 (arrows in Fig. 3a, b), to monitor whether or not those effectively detectable molecules are drifted from the worm body to the outside of body. All four of the molecular ions showed signal intensities that increased by >50 % of the maximum from the inside area of the nematode body (yellow-to-red colors), whereas they appeared near the background level in the area outside of nematode body (blue-to-cyan colors) albeit slightly delocalized to the surrounding glass surface (Fig. 3c). The semi-quantitative analyses of nematodes demonstrated that the averaged signal intensities of all four of the molecules were significantly higher inside than outside the nematode bodies (*n* = 4) (Fig. 3d). These results indicated that the biomolecules were retained inside nematode bodies during the freeze-cracking process.

We further sought to analyze the distribution of biomolecules inside the nematode body with matrix applied via a sublimation deposition method. The averaged mass spectrum acquired from nematode bodies provided a number of mass peaks (Fig. 3e). We selected three of major mass peaks at *m*/*z* 758.4, *m*/*z* 810.4, and *m*/*z* 895.6 to observe the spatial distribution of those molecules in the nematode body. These three molecules showed clearly different distribution patterns in the nematode body: the molecule with *m*/*z* 758.4 was strongly localized at the main body trunk (Fig. 3f; purple); the molecule with *m*/*z* 810.4 was detected almost throughout the nematode body, except in the head and tail regions (Fig. 3f; green); the molecule with *m*/*z* 895.6 was strongly detected in the head and tail regions (Fig. 3f; red).

### Comparative analyses of fatty acid composition of phosphatidylcholine in wild-type and genetically deficient nematodes

**Fig. 4:**
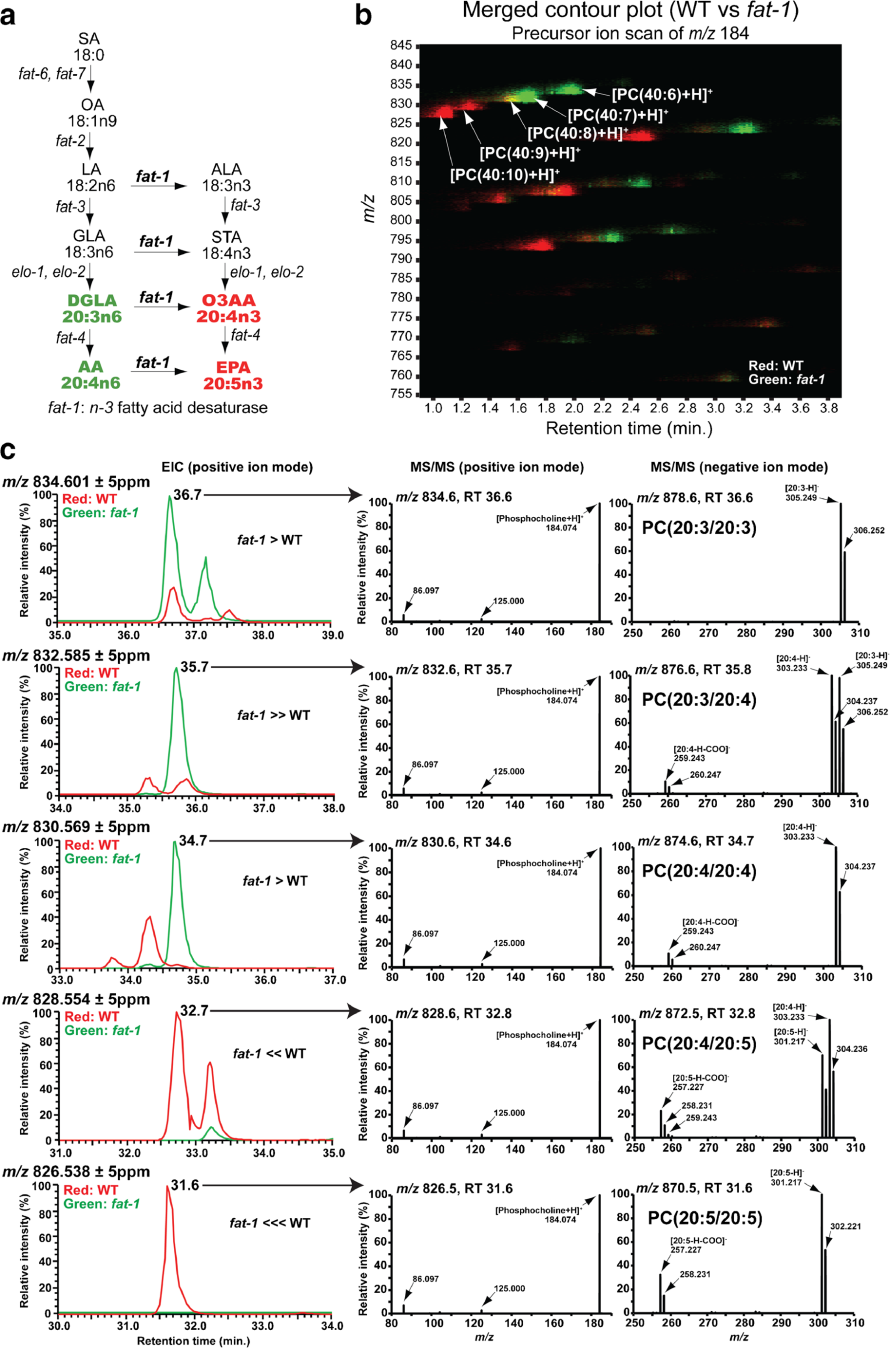
Fatty acid composition of phosphatidylcholine in *fat-1* mutant. **a** Fatty acid desaturation pathway modified from Watts and Browse [23]. *Red*: exclusively detected in the wild type [23]. *Green*: increased in *fat-1* mutants [23]. *SA* stearic acid, *OA* oleic acid, *LA* linoleic acid, *ALA alpha*-linolenic acid, *GLA gamma*-linolenic acid, *STA* stearidonic acid, *DGLA* dihomo-*gamma*-linolenic acid, *O3AA omega*-3 arachidonic acid, *AA* arachidonic acid, *EPA* eicosapentaenoic acid. **b** Merged contour plot obtained by precursor ion scanning of *m*/*z* 184 on lipid extracts from WT and *fat-1*: WT (*red*), *fat-1* mutant (*green*), both (*yellow*). **c** Merged EICs (*left column*): wild-type (*red*) and *fat-1* mutant (*green*). MS/MS spectra at near peak top of picked peaks acquired by targeted MS/MS: In positive ion mode, [M + H]^+^ ions produced a common fragment ion at *m*/*z* 184.074 [phosphocholine + H]^+^ (*middle panel*). In negative ion mode, [M + HCOO]^−^ ions produced fragment ions depending on their fatty acid composition (*right panel*)

We sought to evaluate the potential of our technique for use in combination with nematode genetics for general versatility in analytical biochemistry. For this, we tested the fatty acid composition of PC in the wild-type and genetically deficient *fat-1* mutant nematodes. The *fat-1* mutants lack the gene encoding an *n-3* fatty acid desaturase [23]. Before analyzing the *fat-1* mutants using whole-body MALDI-IMS, we extracted their lipids and profiled the total PC compositions of the two nematode strains by precursor ion scan of *m*/*z* 184, which corresponds to the head group of PC and SM, using LC-ESI-MS/MS. We focused on PC species that contain fatty acids reported to be drastically altered in the *fat-1* mutant (Fig. 4a; red and green) [23], i.e., PC(20:5/20:5), PC(20:4/20:5), PC(20:4/20:4), PC(20:3/20:4), and PC(20:3/20:3). Figure 4b shows a PC ion intensity map (*x*-axis: retention time, *y*-axis: *m*/*z*), in which the PC ion “spots” from the wild-type and *fat-1* mutants were colored red and green, respectively. This merged contour plot showed a clear difference in the PC composition between them (Fig. 4b). Signals at *m*/*z* 828.5 and *m*/*z* 826.5, corresponding to [PC(40:9) + H]^+^ and [PC(40:10) + H]^+^, were almost exclusively detected in wild-type nematodes (Fig. 4b; red). In contrast, signals at *m*/*z* 834.5 and *m*/*z* 832.5, corresponding to [PC(40:6) + H]^+^ and [PC(40:7) + H]^+^, were selectively detected in the *fat-1* mutants (Fig. 4b; green). A signal of *m*/*z* 830.5 corresponding to [PC(40:8) + H]^+^ was detected in comparable quantities in both strains (Fig. 4b; yellow).

We performed further LC-ESI-MS/MS analyses with a hybrid quadrupole-Orbitrap mass spectrometer to determine precise fatty acid compositions of the PC species. PC species were detected as [M + H]^+^ in positive ion mode and [M + HCOO]^−^ in negative ion mode [40]. Figure 4c (left column) shows merged EICs of wild-type (red) and *fat-1* mutant (green) in positive ion mode. EICs corresponding to [M + HCOO]^−^ of each PC species showed almost the same pattern as that of [M + H]^+^ (see Electronic Supplementary Material (ESM) Fig. S1). MS/MS spectra of the most major peak in EICs at each *m*/*z* in the two strains were obtained (Fig. 4c; middle and right columns). MS/MS spectra of minor peaks in EICs were shown in ESM Fig. S2. Consistently with the result of the precursor ion scan, the peaks at *m*/*z* 834.6 (36.6 min), *m*/*z* 832.6 (35.7 min), *m*/*z* 830.6 (34.6 min), *m*/*z* 828.6 (32.8 min), and *m*/*z* 826.5 (31.6 min) in positive ion mode produced a common fragment ion at *m*/*z* 184.0736, which corresponds to [phosphocholine + H]^+^ (Fig. 4c; middle column). MS/MS spectra of negative ion mode showed fragment ion peaks that corresponded to fatty acids (Fig. 4c; right column). PC species containing eicosapentaenoic acid (EPA), PC(20:5/20:5) and PC(20:4/20:5), were almost exclusively detected in wild-type nematodes (Fig. 4c). In contrast, PC species that did not contain EPA, PC(20:4/20:4), PC(20:3/20:4), and PC(20:3/20:3), were detected much more strongly in the *fat-1* mutants than wild type (Fig. 4c).

### Comparative whole-body MALDI-IMS analyses of phospholipid composition in wild-type and genetically deficient nematodes

**Fig. 5:**
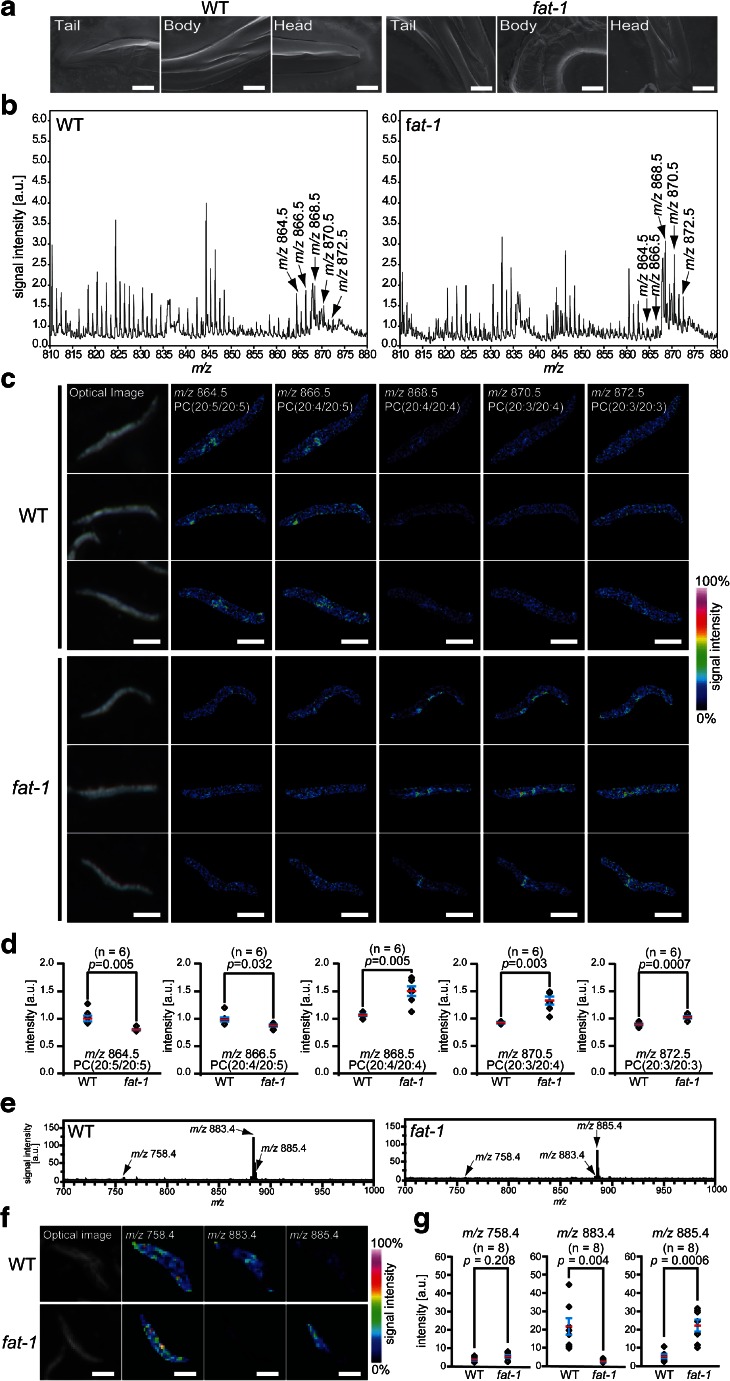
Whole-body MALDI-IMS of genetically deficient nematodes. **a** Scanning electron microscopy images of freeze-cracked wild-type and *fat-1* mutants. Scale bar: 25 μm. **b** Averaged mass spectra detected from freeze-cracked wild-type or *fat-1* mutants in positive ion mode. Five mass peaks at *m*/*z* 864.5, *m*/*z* 866.5, *m*/*z* 868.5, *m*/*z* 870.5, and *m*/*z* 872.5 were selected to depict thermal color scale images. **c** Thermal color scale images of the five selected PC species. Scale bar: 250 μm. Color scale: *deep blue*, faint signal; *red-purple*, maximum signal. **d** Quantitative signal intensities of the PC species in the wild-type and *fat-1* mutants. The data are shown as plots for each nematode (*diamonds*; *n* = 6) and mean (*red line*) ± SD (*blue lines*). The *p* values were calculated using a *t* test. **e** Averaged mass spectra detected from freeze-cracked wild-type or *fat-1* mutants in negative ion mode. Three mass peaks at *m*/*z* 758.4, *m*/*z* 883.4, and *m*/*z* 885.4 were selected to depict thermal color scale images. **f** Thermal color scale images of the three selected mass peaks. Scale bar: 250 μm. Color scale: *deep blue*, faint signal; *red-purple*, maximum signal. **g** Quantitative signal intensities of the detected biomolecular species in the wild-type and *fat-1* mutants. The data are shown as plots for each nematode (*diamonds*; *n* = 8) and mean (*red line*) ± SD (*blue lines*). The *p* values were calculated using a *t* test

With the identified information for the fatty acid compositions of PC molecules in the wild-type and *fat-1* mutants, we performed whole-body MALDI-IMS by applying the freeze-cracking method to both of the strains (Fig. 5a). The averaged mass spectra between *m*/*z* 810–880 included five mass peaks with *m*/*z* values of 864.5, 866.5, 868.5, 870.5, and 872.5 (Fig. 5b), which corresponded to the K^+^ adducts ([M + K]^+^) of the following PC species: PC(20:5/20:5), PC(20:4/20:5), PC(20:4/20:4), PC(20:3/20:4), and PC(20:3/20:3), respectively. The IMS image of each PC species revealed similar PC composition differences between the wild-type and *fat-1* mutants as were detected using LC-ESI-MS/MS (Fig. 5c). PC(20:5/20:5) and PC(20:4/20:5) had higher intensity signals in the wild-type (cyan-to-yellow colors in the thermal color scale) than in the *fat-1* mutants (blue-to-cyan colors) (Fig. 5c). In contrast, PC(20:4/20:4), PC(20:3/20:4), and PC(20:3/20:3) were more abundant in the *fat-1* mutants (cyan-to-orange colors) than in the wild type (blue-to-cyan colors) (Fig. 5c). Semi-quantitative analyses clearly demonstrated the differences of the PC compositions between the wild-type and *fat-1* mutants (*n* = 6) (Fig. 5d), and their statistical values are given in Table 2. The signal intensities of PC(20:5/20:5) and PC(20:4/20:5) were higher in the wild-type than in the *fat-1* mutants (*p* < 0.01 and *p* < 0.05, respectively, *t* test), whereas the signal intensities of PC(20:4/20:4), PC(20:3/20:4), and PC(20:3/20:3) were higher in the *fat-1* mutants than in the wild type (*p* < 0.01 for all cases, *t* test).

**Table 2:** Comparison of signal intensities in the wild-type and *fat-1* mutants

PC; *m*/*z*	Mean signal intensity [a.u.]		Number	*t* test
	WT	*fat-1*	WT/*fat-1*	
PC(20:5/20:5); 864.5	1.05 ± 0.05	0.81 ± 0.001	6/6	<0.01
PC(20:4/20:5); 866.5	1.00 ± 0.04	0.87 ± 0.002	6/6	<0.05
PC(20:4/20:4); 868.5	1.07 ± 0.02	1.50 ± 0.09	6/6	<0.01
PC(20:3/20:4); 870.5	0.93 ± 0.01	1.34 ± 0.008	6/6	<0.01
PC(20:3/20:3); 872.5	0.87 ± 0.02	1.03 ± 0.03	6/6	<0.001

*WT* wild type

We also analyzed the wild-type and *fat-1* mutants in negative ion mode to test the effect of the *fat-1* mutation on phospholipids composition besides PCs. The averaged mass spectra showed only a few peaks (Fig. 5e). The peaks at *m*/*z* 758.4, *m*/*z* 883.4, and *m*/*z* 885.4 were selected to obtain images of those molecules with thermal color scale (Fig. 5f). Two molecules with *m*/*z* 883.4 and 885.4 were detected almost mutually exclusively in wild-type and *fat-1* mutant (Fig. 5f): a molecule with *m*/*z* 883.4 was detected in wild type while a molecule with *m*/*z* 885.4 being detected in *fat-1* mutants (*p* < 0.005, *t* test, *n* = 8) (Fig. 5g). No significant difference was detected for the molecule with *m*/*z* 758.4 (*p* > 0.05, *t* test) (Fig. 5g). These results demonstrated that our whole-body MALDI-IMS technique was capable of being connected to genetics by analyzing individual strains.

## Discussion

We presented a procedure for the direct analysis of the lipid compositions of the whole body of a well-established model organism, *C. elegans*, without requiring lipid extraction or target-specific labeling. We also showed that whole-body MALDI-IMS could offer analyses of molecular distribution in the nematode body and be combined with genetics. The key point of our protocol was to remove the exoskeletal cuticle from nematode bodies, aiming to efficiently generate co-crystals of biomolecules and matrices.

Before this study, two groups tried to directly analyze the components of nematodes without using extraction. One study detected some unidentified biomolecules in larval nematodes using time-of-flight secondary ion mass spectrometry (TOF-SIMS) [41]. This work also combined whole-body imaging with genetics by comparing the molecular compositions of the wild-type and *daf-2* mutants [41]. TOF-SIMS was designed for the analysis of molecules present near the surface of specimens (within several nanometers) [42], and thus would require the removal or bypassing of the cuticle exoskeleton to analyze the intra-body composition. That study thus only detected molecules on the surface of the nematodes, although the cuticle of L1 larvae is thinner than that of adult nematodes [43]. Another study detected manganese, which is not a biomolecule, in larval nematodes using laser ablation-inductively coupled plasma-mass spectrometry (LA-ICP-MS) [44]. LA-ICP-MS was designed for the analysis of elements such as metals, and uses a laser that is >10 times stronger (>1 mJ) [45, 46] than those used in MALDI-IMS (a few hundred μJ). The high-powered laser degrades most organic compounds, although it enables the penetration of the cuticle layer and the ablation of intra-body materials. Furthermore, any degraded fragments of organic compounds would be completely destroyed within the ICP [47]. Thus, LA-ICP-MS is incapable of analyzing organic compounds. Our whole-body MALDI-IMS technique addressed these problems through co-crystallization of phospholipids with a matrix in the nematode body by removing the cuticle using freeze-cracking.

Our technique provided a lipidomic analysis of the PC species in genetically deficient mutant nematodes. The result of the comparative whole-body IMS between the wild-type and *fat-1* mutants is consistent with the fatty acid compositions reported previously [23–25]. The lower level of EPA-containing PCs, PC(20:5/20:5) and PC(20:4/20:5), in *fat-1* mutants is consistent with the finding that they lose EPA [24]. The higher level of dihomo-*gamma*-linolenic acid (DGLA)-containing PCs, PC(20:3/20:4) and PC(20:3/20:3), in *fat-1* mutants is consistent with the finding that DGLA is accumulated in them [24]. These results were also self-consistent with our own LC-ESI-MS/MS analyses. The higher level of PC(20:4/20:4) in *fat-1* mutants is also consistent with the past finding that the amount of fatty acid (20:4) is higher in *fat-1* mutants than the wild type [24]. Given that *omega*-3 arachidonic acid (O3AA; 20:4n3) was the major fatty acid (20:4) in the wild type and AA (20:4n6) was the sole fatty acid (20:4) in the *fat-1* mutants [24], PC(20:4/20:4) in the wild type could be PC(20:4n3/20:4n3), whereas that in the *fat-1* mutants could be PC(20:4n6/20:4n6). Notably, PC(20:4/20:4) was detected at different retention time in the wild-type (33.7 min) and the *fat-1* mutants (34.7 min). This highlights the caution required in data interpretation of MALDI-IMS in cases where molecules with identical *m*/*z* values, but different structures, are compared, since the ionization efficiency in MALDI-IMS seems to differ based on molecular structure [48]. Some parts of our data might require the caution. The molecule with *m*/*z* 868.5 assigned as PC(20:4/20:4) in MALDI-IMS could be PC(20:3/20:5) in the wild type, and the molecule with *m*/*z* 872.5 assigned as PC(20:3/20:3) in MALDI-IMS could contain the fewer level of PC(20:2/20:4), based on the LC-ESI-MS/MS data. This structure-dependent complexity could be resolved more clearly when using IMS by means of ion mobility mass spectrometry in the future.

Our MALDI-IMS data in negative ion mode provided a clear difference of lipid composition between the wild-type and *fat-1* mutants. The molecule with *m*/*z* 883.4 almost exclusively detected in the wild type is highly likely to be a PI containing EPA, PI(18:0/20:5), since PI(18:0/20:5) is the most major PI form in normal *C. elegans* [49, 50]. This idea is consistent with the knowledge that *fat-1* mutants lose EPA [24] and our findings that EPA-containing PCs were detected predominantly in the wild type. The molecule with *m*/*z* 885.4 is highly possible to be PI(18:0/20:4), which is rare in normal *C. elegans* [49, 50] while being normally detected and most major in mammalian tissues [51]. This idea is also consistent with the finding that AA is abnormally accumulated in *fat-1* mutants [24] and our findings that PCs, PC(20:3/20:4) and PC(20:4/20:4), were detected almost exclusively in *fat-1* mutants.

Our technique could offer two additional benefits in the metabolomics analyses of nematodes. First, the technique provides spatial information regarding the uneven distribution of different biomolecules in individual nematode bodies. Rough sketches of molecular distribution could be determined in nematodes, as our IMS analyses combined with the matrix sublimation technique provided different mutually exclusive distribution of different molecules in a nematode body. Second, the technique could analyze multiple individuals in a single experiment. This has great potential for the comparison of individual-to-individual variations in molecular composition and metabolic responses to environmental stimuli. Taken together, our technique provides the potential to perform multi-dimensional omics analyses including the time-dependent alteration of metabolite levels, at both the intra-individual and inter-individual levels.

## Electronic supplementary material

ESM 1 (PDF 11703 kb)
