# Supplementary material for: Direct profiling of the phospholipid composition of adult Caenorhabditis elegans using whole-body imaging mass spectrometry
Source: Anal Bioanal Chem. 2015 Aug 27;407(25):7589–602. doi: 10.1007/s00216-015-8932-7 (PMC4575384; doi:10.1007/s00216-015-8932-7)
Supplement: Supplementary file 1 — (PDF 11703 kb) [file 216_2015_8932_MOESM1_ESM.pdf]

Analytical and Bioanalytical Chemistry

Electronic Supplementary Material

Direct profiling of the phospholipid composition of adult *Caenorhabditis elegans* using whole-body imaging mass spectrometry

Saira Hameed, Koji Ikegami, Eiji Sugiyama, Shoko Matsushita, Yoshishige Kimura, Takahiro Hayasaka, Yuki Sugiura, Noritaka Masaki, Michihiko Waki, Isao Ohta, Md Amir Hossen, Mitsutoshi Setou

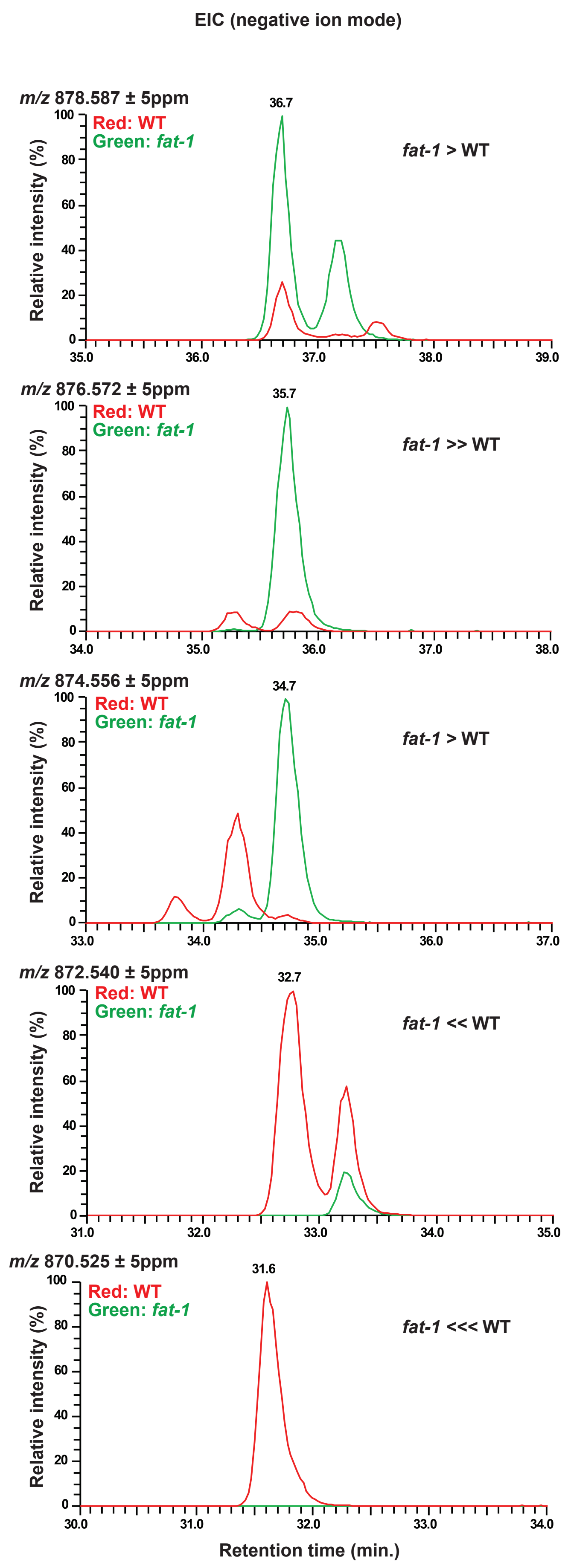

Fig. S1 Merged EICs acquired through the scanning between *m/z* 700 to 900 by a hybrid quadrupole-Orbitrap mass spectrometer in negative ion mode. Red, WT; green, *fat-1* mutant.

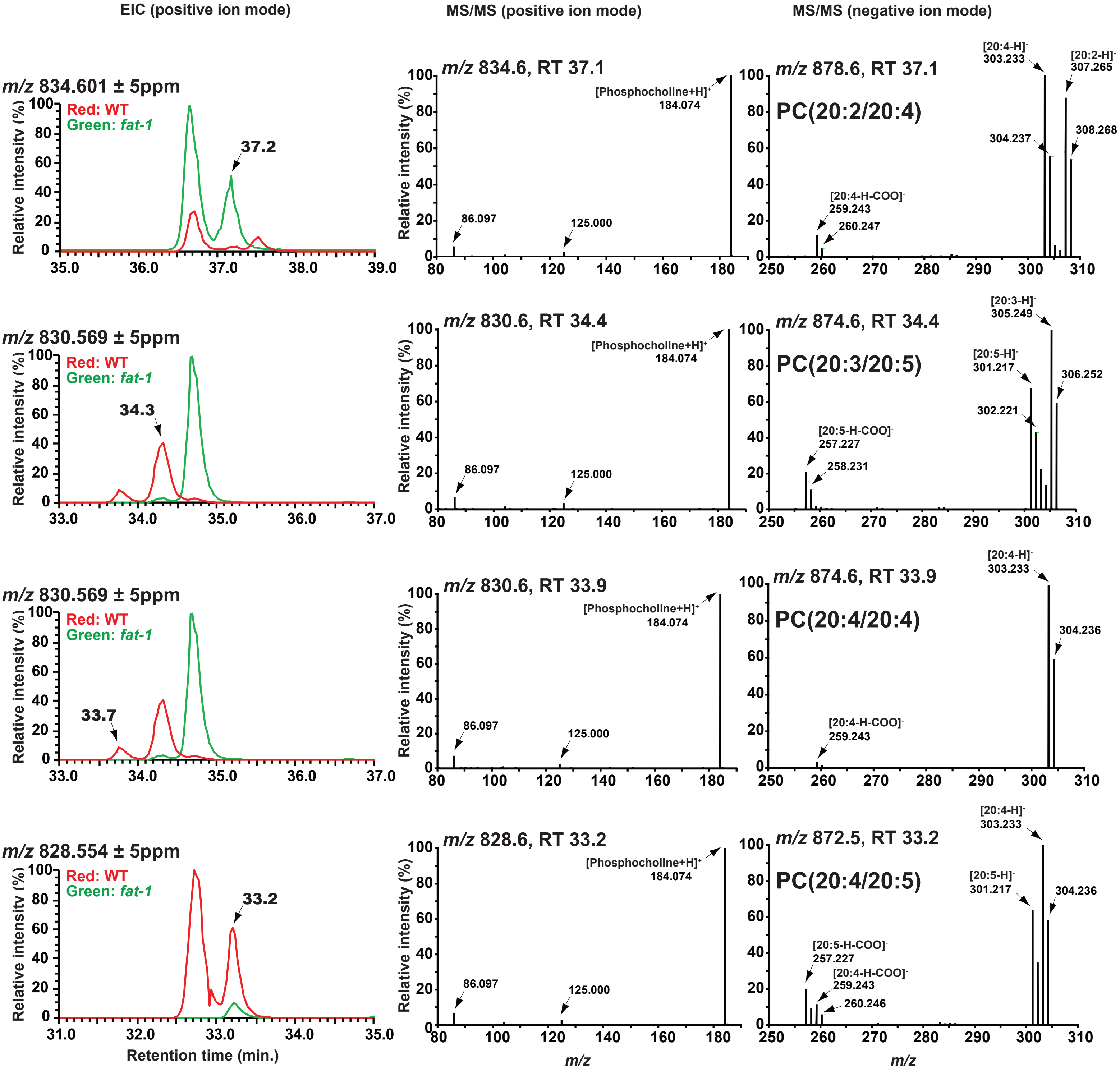

Fig. S2 (Left collumn) Merged EICs acquired through the scanning between *m/z* 700 to 900 by a hybrid quadrupole-Orbitrap mass spectrometer. Red, WT; green, *fat-1* mutant. (Middle collumn) MS/MS spectra at near peak top of picked peaks acquired by targeted MS/MS in positive ion mode. $[M+H]^+$ ions produced a common fragment ion at *m/z* 184.074 $[phosphocholine+H]^+$. (Right collumn) MS/MS spectra at near peak top of picked peaks acquired by targeted MS/MS in negative ion mode. $[M+HCOO]^-$ ions produced fragment ions depending on their fatty acid composition.
